# Alcohol and the Hormonal Control of Lactation

**Published:** 1998

**Authors:** Sarah H. Heil, Marappa G. Subramanian

**Affiliations:** Sarah H. Heil, Ph.D., is a postdoctoral fellow in the Department of Obstetrics and Gynecology and Marappa G. Subramanian, Ph.D., is a professor in the Department of Obstetrics and Gynecology and in the Department of Physiology and is director of operations of The C. S. Mott Center for Human Growth and Development, Department of Obstetrics and Gynecology, Wayne State University School of Medicine, Detroit, Michigan

**Keywords:** AODE (alcohol and other drug effects), mammary gland, lactation, breast milk, gestation, prolactin, oxytocin, ethanol metabolism, excretion, prenatal alcohol exposure, animal model, mother, offspring, literature review

## Abstract

All mammals produce milk to nourish their young. Milk production (i.e., lactation), which occurs in the mammary glands, is regulated by several hormones, most prominently prolactin and oxytocin. Studies in both humans and laboratory animals have demonstrated that maternal alcohol consumption before and during lactation can interfere with the functions of both of those hormones. Moreover, animal studies found that maternal alcohol consumption during pregnancy and even earlier in the mother’s life can impair mammary gland development. Maternal alcohol consumption during pregnancy and lactation also can alter the milk’s nutrient composition and result in suckling deficits of the offspring. Alcohol (and possibly its breakdown products) can pass from the maternal circulation into the breast milk. The effects of these substances on the infant, however, are still unknown.

Milk production (i.e., lactation) is a major component of reproduction in mammals. In contrast to all other animals, mammals—including primitive mammals, such as the duckbill platypus and the spiny anteater—nourish their offspring with a liquid (i.e., milk) that is secreted by specialized glands (i.e., the mammary glands). Milk production and secretion are complex, multistage processes that are controlled by several hormones, the most important of which are prolactin and oxytocin. The major stages involved in lactation include the structural development of the mammary glands, the initiation of milk secretion at the birth (i.e., parturition) of the offspring, and the maintenance of postpartum milk production.

The milk serves two important functions. The so-called foremilk, which is initially produced after parturition, helps protect the infant offspring against disease during the initial days after birth. The foremilk contains large amounts of antibodies—molecules produced by the mother’s immune system that can attack disease-causing microorganisms and initiate the immune response. The primary function of the “mature milk,” which subsequently is produced for the duration of lactation, is to nourish the offspring.

Alcohol exerts its multiple effects on reproduction, including lactation, mainly by influencing the secretion and function of numerous hormones. This article briefly summarizes the processes involved in normal mammary gland development as well as in the initiation and maintenance of lactation. The article then reviews studies that have investigated alcohol’s effects on animal and human maternal milk production and on the offsprings’ nursing behavior.

## Lactation

### Mammary Gland Development

**Figure 1:**
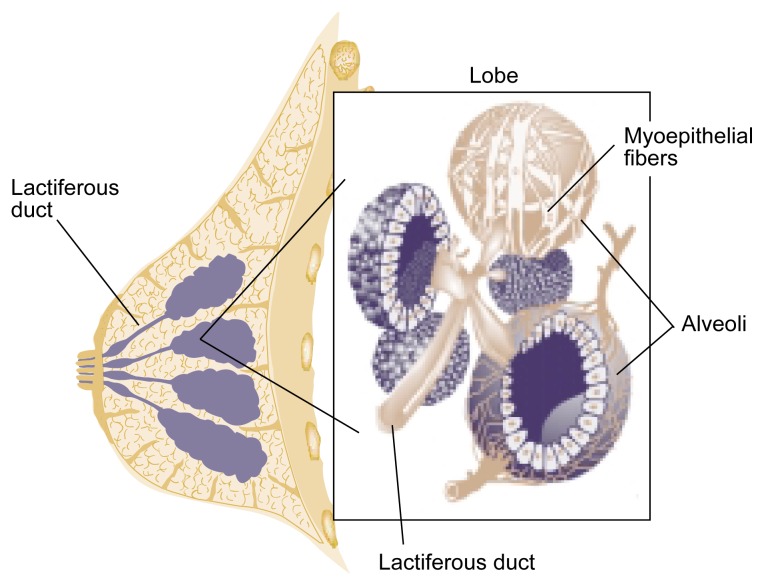
Structure of the female breast and mammary glands. The milk-secreting cells form ball-shaped structures, the alveoli, which are covered on the outside by myoepithelial cells. Several alveoli combine to form a lobule, although a lobule may contain as little as one alveolus and its duct (i.e., the lobular duct). Several lobules form a lobe. The ducts of the lobes (i.e., lactiferous ducts) converge at the nipple. SOURCE: Adapted from Cunningham, F.G.; MacDonald, P.C.; Gant, N.F.; Leveno, K.J.; and Gilstrap, L.C., III (eds.). *Williams Obstetrics*. 19th ed. Norwalk, CT: Appleton and Lange, 1993.

Mammary glands are modified skin (i.e., cutaneous) glands found in all female mammals. The glands consist of numerous alveoli—structures that contain the milk-secreting cells (see figure 1). Each alveolus releases the secreted milk into a small duct. Several alveoli and their ducts form a structural unit called a lobule. The milk secreted by the lobules is collected in an interlobular duct. Several lobules combine to form a lobe. Within each lobe, the interlobular ducts converge into a lactiferous duct, which transports the milk to the nipple. In humans, each breast contains 15 to 20 lobes.

**Figure 2:**
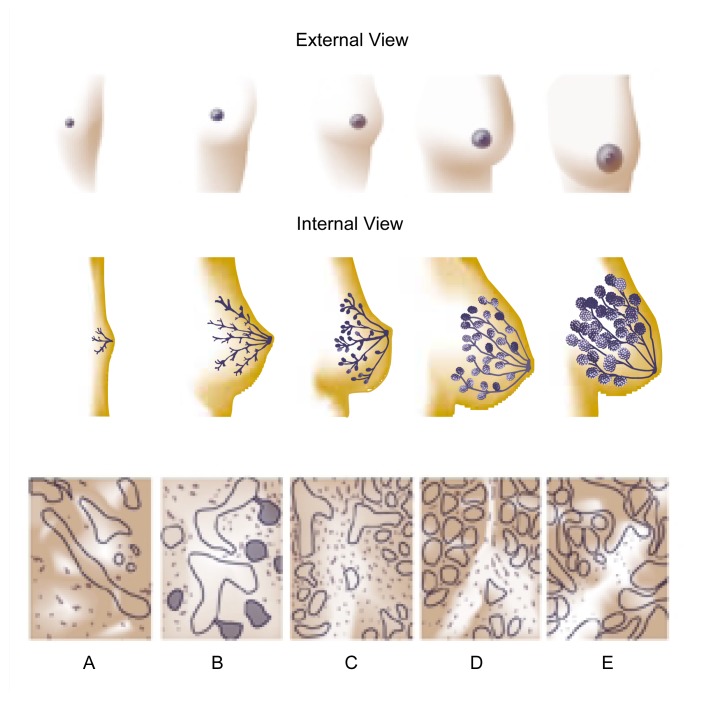
Development of the female breast and mammary glands: (A) during infancy, (B) before puberty, (C) after puberty, (D) during pregnancy, and (E) during lactation. SOURCE: Adapted from Lawrence, R.A. Breastfeeding: A Guide for the Medical Profession. St. Louis: Mosby, 1994.

Mammary gland development begins early in fetal life and continues throughout gestation and after birth. Although the glands grow somewhat around puberty, the greatest morphological changes in mammary gland architecture occur during pregnancy (see figure 2, p. 180). A detailed description of the development and differentiation of the mammary gland before the initiation of lactation is beyond the scope of this article; the reader should refer to earlier reviews by Tucker (1979) and Whitworth (1988).

Numerous hormones help regulate mammary gland growth and differentiation, including hormones produced by the anterior pituitary gland (i.e., prolactin and growth hormone), thyroid gland (i.e., thyroxine), adrenal glands (i.e., glucocorticoids), ovaries (i.e., estrogen and progesterone), and pancreas (i.e., insulin). (For more information on these hormones and their functions, see the article by Hiller-Sturmhöfel and Bartke, pp. 153–164.) In addition, the fetus and its part of the placenta (i.e., the fetoplacental unit) function as a hormone-producing (i.e., endocrine) organ during late pregnancy, secreting several steroid hormones that affect mammary gland development, such as estrogen and progesterone.

The relative contributions of various hormones and the sequence of events in mammary gland development are difficult to assess. Detailed studies in rodents have established that estrogen is essential for the growth of the interlobular and lactiferous ducts. Moreover, both estrogen and progesterone contribute to duct growth and to the development of the lobules and alveoli. In addition to estrogen and progesterone, the pituitary hormones prolactin and growth hormone are required for complete mammary gland development. The other hormones listed above (i.e., thyroxine, glucocorticoids, and insulin) are required for optimal metabolic function of the mammary gland (Whitworth 1988). The results obtained in rodents may not be completely transferable to human mammary gland development, however, because the roles of specific hormones can vary among different species.

### Initiation of Lactation

During pregnancy, the development of the mammary gland secretory apparatus is completed under the influence of increasing levels of prolactin, placental lactogen, estrogen, and progesterone. Although prolactin levels rise throughout pregnancy, not all the hormone’s actions (e.g., the initiation of lactation) occur at that time, because the high levels of estrogen and progesterone prevent some of prolactin’s effects. Following parturition, however, progesterone and estrogen levels decline. As a result, prolactin can exert its effects in the mammary tissue and initiate milk secretion.

### Maintenance of Lactation

Prolactin is essential not only for the initiation of lactation after parturition but also for the maintenance of lactation. In response to the infant’s suckling at the breast, a surge of prolactin is released from the pituitary gland. This suckling-induced prolactin release is essential for the mammary gland to produce sufficient milk before the next feeding. The intensity of the suckling affects both the magnitude and the pattern of the increase in prolactin levels in the blood. Between feedings, when no suckling occurs, however, prolactin levels are not elevated.

In addition to prolactin, successful lactation also requires the hormone oxytocin.^1^ The hormone promotes milk ejection and the emptying of the breast. Oxytocin also is released from the pituitary gland into the blood, although from a different area (i.e., the posterior pituitary gland). As with prolactin, oxytocin release occurs in response to a suckling stimulus. Oxytocin causes certain cells (i.e., myoepithelial cells) surrounding the alveoli and alveolar ducts to contract and to expel the secreted milk into the interlobular and lactiferous ducts (see figure 1). This reaction, which occurs rapidly after the initiation of suckling, is called the let-down reflex. Short (1984) has summarized the actions of oxytocin and prolactin as follows: “Oxytocin serves today’s meal and prolactin prepares tomorrow’s” (p. 36).

## Alcohol and Lactation

As indicated in the previous section, effective lactation depends on various factors, including appropriate control by prolactin and oxytocin, intact mammary glands that can produce a sufficient quantity and quality of milk, and effective suckling by the infant. Maternal alcohol consumption before and during lactation can interfere with each of these factors. The following sections summarize current knowledge regarding alcohol’s effects on lactation, as determined by studies in humans and laboratory animals.

### Alcohol’s Effects on the Hormones Influencing Lactation

#### Prolactin Secretion

Although numerous hormones are involved in the lactational process, prolactin is the key hormone controlling milk synthesis. During lactation, the infant’s suckling at the breast leads to the generation of nerve signals that are transmitted by sensory nerves to the hypothalamus in the brain. From there, the signals are conveyed to the anterior pituitary gland, where they induce prolactin release.

Alcohol’s effects on prolactin release have not been studied in lactating women. In a study conducted in non-lactating women, however, alcohol consumption that resulted in a blood alcohol level (BAL) of approximately 0.08 percent^2^ diminished prolactin secretion in response to breast stimulation (Volpi et al. 1994). Other researchers have investigated the effects of both acute and chronic alcohol administration on prolactin secretion and other lactational variables in lactating rats. Those studies have demonstrated that neither acute nor chronic (8-day) alcohol administration affects basal prolactin levels in lactating rats that are separated from their pups (Subramanian 1996). The suckling-induced surge in prolactin release, in contrast, is inhibited by both acute and chronic alcohol administration. Other experiments have indicated that alcohol administered after a suckling-induced prolactin surge has been established inhibits further prolactin release; however, the inhibition can be overcome if the pups continue nursing for 120 minutes (Subramanian 1991). This effect resulted from the continued suckling, because the BAL remained elevated during the experimental period. Finally, a series of studies have demonstrated that alcohol does not act at the pituitary level to inhibit suckling-induced prolactin release (Subramanian 1996). This observation suggests that alcohol may inhibit suckling-induced prolactin release either by acting at the level of the hypothalamus or other brain structures or by disrupting the transmission of nerve signals generated at the nipples in response to suckling.

#### Oxytocin Secretion

The second major hormone involved in lactation is oxytocin. Early studies investigating alcohol’s effects on oxytocin’s functions in lactating rabbits, rats, and humans indicated that alcohol reduced uterine contractions (Fuchs 1969). Those findings imply that alcohol inhibits oxytocin release. Cobo (1973) also demonstrated that alcohol doses of up to 2.0 grams per kilogram body weight, which resulted in BALs as high as 0.3 to 0.4 percent, inhibited milk ejection in lactating women in a dose-dependent manner. Finally, alcohol has been shown to inhibit not only prolactin release but also oxytocin release in response to breast stimulation in nonlactating women (Coiro et al. 1992). As with prolactin release, several mechanisms may account for alcohol’s effect on oxytocin release. Those potential mechanisms include actions at the level of the hypothalamus or other brain structures and disruption of the transmission of the nerve impulses engendered at the nipples.

### Alcohol’s Effects on the Mammary Gland and Milk

#### Effects on the Mammary Gland

Several studies have indicated that alcohol can significantly influence the lactation process by altering mammary gland development. For example, in rats that received drinking water with 5-percent alcohol during the second half of pregnancy, the mammary gland weighed less and appeared to secrete milk less efficiently than in untreated rats. When the alcohol was given from parturition throughout lactation, however, no effects on the mammary gland were observed (Jones and Stewart 1984). Furthermore, Steven and colleagues (1989) demonstrated that alcohol administration beginning 25 days before mating and lasting through pregnancy up to day 2 or day 10 of lactation altered the mammary gland structure during the early stages of lactation. A shorter duration of alcohol administration (i.e., from day 1 of pregnancy to day 2 or day 10 of lactation), in contrast, did not result in mammary tissue changes. These observations indicate that chronic alcohol ingestion can interfere with lactation, particularly if it occurs during or before pregnancy. Differences in the methodology between the studies of Jones and Stewart (1984) and Steven and colleagues (1989), such as in the mode of alcohol administration (i.e., in the drinking water versus as a liquid diet) and in the alcohol dose (i.e., 5 percent versus 6 percent) likely account for the discrepancies in the results of those two studies.

Other studies in rats also demonstrated that alcohol exposure during the early postnatal period could interfere with normal mammary gland development later in life. Singletary and McNary (1992) administered alcohol to 21- to 22-day-old rats for 32 days and compared the mammary gland development in those animals with that of control rats that had not received alcohol. The study found that in the alcohol-fed rats, the maturation of the developing mammary gland was delayed and that structures which undergo differentiation during this stage were altered. In addition, the alcohol treatment resulted in lower progesterone levels shortly before the animals went into heat (i.e., during proestrus). This stage of the estrous cycle is characterized by increased serum levels of progesterone, estradiol, and other sex hormones. In young animals, such as those used in this study, increased progesterone levels are essential for the maturation of the mammary cells into alveoli structures.^3^ These observations suggest that alcohol’s adverse effect on mammary gland development may be related to the alcohol-induced reduction in progesterone levels.

#### Alcohol’s Effects on Milk Constituents

To fulfill its purpose of adequately nourishing the offspring, milk normally consists of a balanced mixture of nutrients, including sugars (primarily lactose), proteins (primarily casein), fats (i.e., lipids), minerals, and vitamins. Several animal studies have indicated that chronic alcohol consumption may alter the milk’s composition as follows:

- In female rats that received alcohol in their drinking water starting 4 weeks before mating and lasting through pregnancy and into the latter part of lactation, casein production and secretion in the milk were impaired, although lipid production and secretion remained unaffected (Vilaro et al. 1989).
- In the milk of rats that had been treated chronically with alcohol starting 4 to 5 weeks before mating, the levels of proteins and lipids increased and lactose levels decreased compared with untreated control rats (Sanchis and Guerri 1986). In addition, the milk became more alkaline in the alcohol-treated animals. These findings contradict those of Vilaro and colleagues (1989) described above. Differences in methodology of the two studies (i.e., different modes of alcohol administration and different alcohol doses used) likely account for the discrepancies.
- Chronic alcohol administration interfered with the uptake of the building blocks of proteins (i.e., amino acids) by the mammary glands (Vinas et al. 1987). The extent of this adverse effect varied, however, depending on the amino acid studied.
- Alcohol administration in the drinking water for 4 weeks before mating, during pregnancy, and through lactation adversely affected numerous indicators of mammary gland function (Vilaro et al. 1987). For example, overall milk production declined, as did the milk’s protein and lactose contents. Moreover, lipoprotein lipase, an enzyme that breaks down molecules consisting of proteins and lipids (i.e., lipoproteins), showed increased activity. Consistent with this observation, triacylglycerol, which is a breakdown product of many lipids and lipoproteins, increased in concentration. Finally, both the absolute and the relative weight of the mammary glands decreased, although the gland’s dry weight increased, suggesting incomplete alveolar development.
- The levels of a certain lipid component (i.e., phosphatidylserine) were elevated in the milk of animals that received alcohol on days 2 through 16 of lactation (Hungund et al. 1996). In addition, cholesterol levels in the blood of the alcohol-treated animals were elevated (Heil et al. 1998).

Furthermore, the results of a recent study in lactating women suggest that after ingestion of a small dose of alcohol (0.3 gram per kilogram body weight) in orange juice, the women produced less milk than after consuming orange juice alone (Menella 1998). Taken together, the findings indicate that alcohol consumption before and during lactation can interfere with the lactational process by compromising milk quality and quantity, thereby interfering with the optimal nourishment of the offspring.

#### Alcohol Excretion in Milk

Several factors influence how much of a given drug, such as alcohol, will pass from the maternal circulation into the breast milk. Those factors include the pharmacokinetic properties of the drug and its breakdown products as well as the drug’s solubility in water, pH, molecular weight, and degree with which it binds to proteins (Lawrence 1994). Alcohol passes freely from the mother’s blood into the breast milk. It is also well established that the alcohol concentration in human milk is similar to that in the blood and that alcohol’s elimination from blood and milk is closely correlated (Lawton 1985). The blood alcohol levels that result in nursing babies whose mothers have consumed alcohol are thought to be very low, however, because the alcohol contained in the milk would be substantially diluted by the baby’s body water (Lawton 1985). For example, Wilson and colleagues (1980) calculated that an infant whose mother had a BAL of 0.1 percent would receive 164 milligrams of alcohol during a single feeding. Although this constitutes a small amount, it is unknown what effect, if any, such an alcohol dose might have in an infant. Little and colleagues (1989) reported that 12-month-old infants whose mothers had regularly consumed alcohol (at least one drink daily) while nursing exhibited a small but significant reduction in gross motor development, even when the women’s alcohol consumption during pregnancy was controlled for. No significant correlation existed, however, between maternal alcohol consumption during lactation and the infant’s mental development.

Another concern regarding alcohol consumption in nursing women is whether the major breakdown product of alcohol, acetaldehyde, can pass into the milk. Acetaldehyde has been suggested to be responsible for much of the alcohol-induced physiological damage in the drinker. The findings concerning the transfer of acetaldehyde into the milk are inconsistent. Kesaniemi (1974) detected no acetaldehyde in the milk after alcohol consumption, although significant acetaldehyde amounts were present in the mother’s blood. In contrast, Guerri and Sanchis (1986) used rats that had been administered alcohol to demonstrate that acetaldehyde concentrations in the milk reached 35 to 50 percent of the concentrations found in the blood. Differences in methodology, particularly in the subject population (i.e., humans versus rats), may account for the different results of the studies by Kesaniemi (1974) and Guerri and Sanchis (1986). Given the discordant results, it is difficult to draw any conclusions about the potential effects of acetaldehyde on infants.

### Alcohol Exposure and Suckling Deficits in the Offspring

As mentioned earlier in this article, suckling by the offspring is crucial for the pituitary gland’s release of prolactin and oxytocin, which, in turn, influence milk synthesis and release. Consequently, suckling deficits induced by maternal alcohol use during the prenatal and/or postnatal periods might alter hormone release and subsequent milk production and secretion.

#### Effects of Prenatal Alcohol Exposure

Infants born with fetal alcohol syndrome resulting from their mothers’ prenatal alcohol abuse exhibit severe feeding dysfunction. Those infants suckle poorly, tire quickly while feeding, and are easily distracted (Van Dyke et al. 1982). Rat pups prenatally exposed to alcohol attach to the nipple more slowly; exert a lower maximum suckling pressure; spend less time suckling; and perform fewer rapid, rhythmic sucks per suckling time period compared with pups that have not been exposed to alcohol (Rockwood and Riley 1986). Similarly, Subramanian (1992) demonstrated that rat pups that had been exposed to alcohol prenatally required more time to attach to the nipple and exhibited decreased milk consumption as well as a decreased growth rate.

The effects of alcohol-induced suckling deficiencies on prolactin release were investigated using alcohol-exposed rat pups and non–alcohol-exposed foster mothers (Subramanian 1992). In the foster mothers, prolactin release in response to suckling by prenatally alcohol-exposed pups showed an interesting pattern. On day 6 of lactation, no difference in prolactin secretion existed between foster mothers nursing alcohol-exposed pups and foster mothers nursing control pups. On day 10 of lactation, however, the suckling-induced prolactin release was enhanced in foster mothers of alcohol-exposed pups. This somewhat paradoxical response may represent a compensatory hormonal mechanism in the foster mothers to ensure adequate milk secretion in response to the poor suckling. Alternatively, the increased prolactin release may result from shorter but more frequent suckling episodes by alcohol-exposed pups. The latter explanation is consistent with the hypothesis that optimal lactation depends on the frequency, rather than the duration, of suckling (Short 1984).

#### Effects of Postnatal Alcohol Exposure

Popular folklore has suggested that alcoholic beverages may have a beneficial effect on lactation by helping the mother relax before feeding and thereby promoting the let-down reflex. In contrast to those assumptions, however, recent studies found that breast-fed infants consumed less milk during a test session after their mothers drank beer or a small dose of alcohol in orange juice (Menella in press). In animal studies, rat pups whose mothers received alcohol for as little as 4 days required more time to attach to the nipple (Subramanian et al. 1990) and exhibited decreased milk consumption (Subramanian 1997). Those observations indicate that both prenatal and postnatal alcohol exposure adversely affect suckling and may thereby contribute to postnatal growth retardation and behavioral problems.

## Summary

Alcohol is a widely available and abused social drug. Although popular folklore suggests that alcohol consumption may not be detrimental to a lactating woman and her infant and may actually benefit the mother, the medical community continues to debate alcohol’s effects on lactation. For example, a 1987 letter published in the *Journal of the American Medical Association* that asked whether any scientific basis existed for recommending drinking one beer per day to a lactating woman engendered numerous and varied responses (Menella in press). A substance abuse specialist recommended avoiding the prescription of alcohol because it can be considered a drug. A lactation consultant and an obstetrician suggested that alcohol may be an appropriate recommendation if the mother also receives instructions about the appropriate timing and alcohol dose. Finally, a pediatrician proposed that one drink per day probably would not cause harm. A report of the Committee on Drugs of the American Academy of Pediatrics (1994) classified alcohol as a drug compatible with breast feeding. The Committee also recognized, however, that consumption of large amounts of alcohol may lead to adverse effects, such as decreases in linear growth and abnormal weight gain in the infant and a decreased let-down reflex in the mother. These divergent opinions indicate that more research is required to delineate the exact effects of alcohol on lactation.

Animal studies have indicated that alcohol consumption, particularly over a longer period of time, can adversely affect all stages of the lactational process. For example, through its effects at the level of the hypothalamus and/or pituitary gland, alcohol may modulate the levels or activities of hormones that govern mammary gland development as well as initiation and maintenance of lactation. Chronic alcohol consumption also may affect the quantity as well as quality of various milk constituents that are necessary to ensure optimal nourishment of the offspring. Finally, alcohol’s effects on the offspring’s suckling behavior also could lead to changes in hormone levels in the mother and, subsequently, in the quantity of milk produced.

Recent studies in human volunteers have added to researcher’s understanding of alcohol’s effects on lactation. Additional analyses using human volunteers, combined with the recent development of new, more sensitive investigative tools, will allow researchers to further explore the specific mechanisms and sites of action of alcohol on hormonal, nutritional, and other factors controlling lactation.

## Footnotes

1 In addition to its function during lactation, oxytocin stimulates the contraction of the uterine muscles during labor.

2 The women drank 110 milliliters (mL) of whiskey containing 50 mL of pure alcohol. This dose resulted in a mean peak BAL of 78 milligrams per deciliter (mg/dL ) (0.078 percent). In the United States, the legal BAL limits for driving are between 0.08 and 0.1 percent.

3 In the sexually mature rat, progesterone is essential during the proestrus to induce a rapid surge of luteinizing hormone from the pituitary gland, which in turn triggers ovulation.
